# Supplementary material for: Comparison of Commercially Available Thermostable DNA Polymerases with Reverse Transcriptase Activity in Coupled Reverse Transcription Polymerase Chain Reaction Assays
Source: Methods Protoc. 2025 Jan 26;8(1):11. doi: 10.3390/mps8010011 (PMC11858481; doi:10.3390/mps8010011)
Supplement: Supplementary file 1 [file mps-08-00011-s001.zip › Smirnova_et_al_Suppl/FigureS1.pdf]

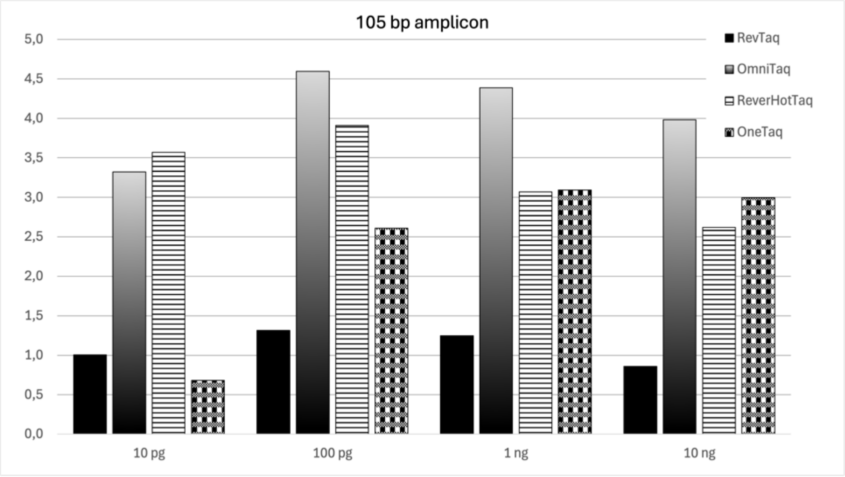

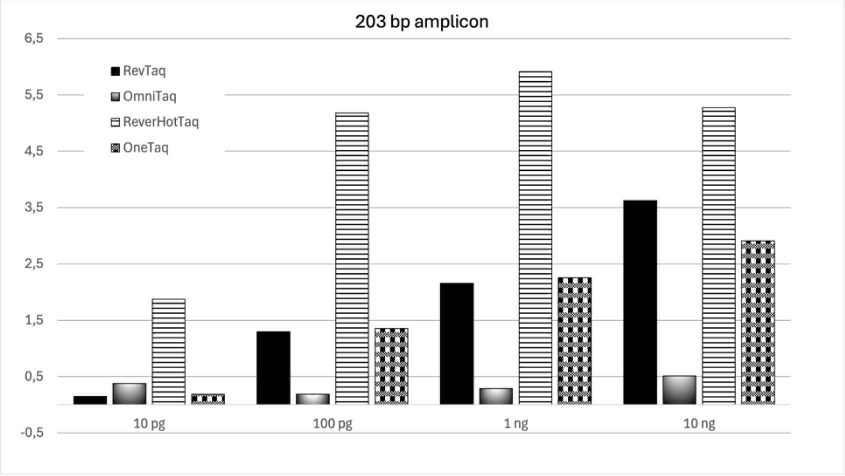

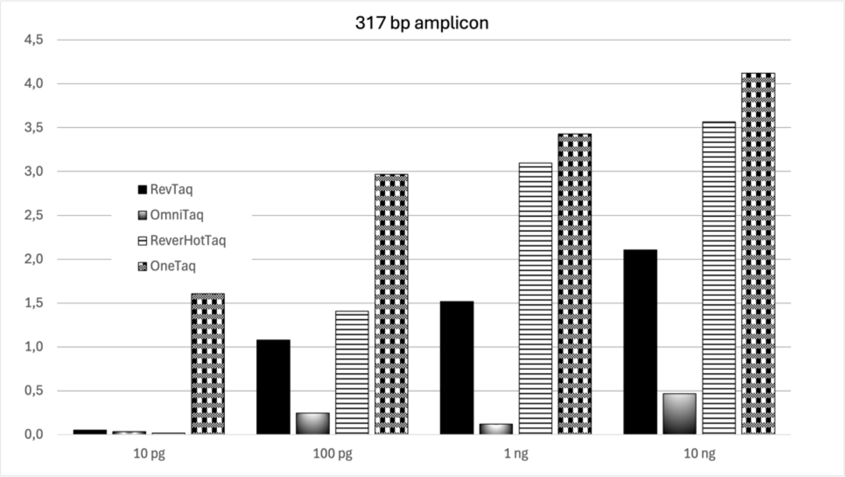

Figure S1. Densitometry analysis of the amplicons from the end-point RT-PCR assays with RevTaq, OmniTaq 2, and ReverHotTaq DNA polymerases and OneTaq One-Step Enzyme Mix. The Mean grey values of the bands determined by ImageJ program were backgrounded and normalized to the corresponding MW marker bands (raw data is provided in the Table S1). Series correspond to the enzymes used, Y-axis is the relative intensity, X-axis is the template amount.
